# Mapping of health technology assessment in China: a comparative study between 2016 and 2021

**DOI:** 10.1186/s41256-023-00339-6

**Published:** 2024-01-16

**Authors:** Shimeng Liu, Yu Xia, Yi Yang, Jian Ming, Hui Sun, Yan Wei, Yingyao Chen

**Affiliations:** 1 School of Public Health, Fudan University, Shanghai, 200032 China; 2 National Health Commission Key Laboratory of Health Technology Assessment (Fudan University), Shanghai, 200032, China; 3 Shanghai Health Development Research Center, Shanghai, 201199 China

**Keywords:** Health technology assessment, Mapping, Development level, Propensity score matching, China

## Abstract

**Background:**

Health Technology Assessment (HTA) in China has recently expanded from purely academic research to include policy or decision-oriented practice, especially after HTA evidence was used to update the National Reimbursement Drug List for the first time in 2017. This study aims to identify the progress and challenges of HTA development from 2016 to 2021 and inform policies and decisions to promote further HTA development in China.

**Methods:**

We conducted a cross-sectional web-based survey with policy makers, researchers and industry-providers in China in 2016 and 2021 respectively. The ‘Mapping of HTA Instrument’, was utilized to assess the HTA development across eight domains: Institutionalization, Identification, Priority setting, Assessment, Appraisal, Reporting, Dissemination of findings and conclusions, and Implementation in policy and practice. To reduce the influence of confounders and compare the mapping outcomes between the 2016 and 2021 groups, we conducted 1:1 Propensity Score Matching (PSM). Univariate analysis was conducted to compare the differences between the two groups. The overall results were further compared with those of a mapping study that included ten countries.

**Results:**

In total, 212 and 255 respondents completed the survey in 2016 and 2021, respectively. The total score of the HTA development level in China in 2021 was higher than that in 2016 before PSM (89.38 versus 83.96). Following PSM, 183 respondents from the 2016 and 2021 groups were matched. Overall, the mean scores for most indicators in the Institutionalization domain and Dissemination domain in 2021 were higher than those in 2016 (*P* < 0.05). The Appraisal domain in 2021 was more explicit, transparent and replicable than that in 2016 (*t* = −3.279, *P* < 0.05). However, the mean scores of most indicators in the Assessment domain were higher in 2016 than those in 2021 (*P* < 0.05).

**Conclusions:**

Our study suggest that the level of HTA development in China progressed significantly from 2016 to 2021. However, before engaging in HTA activities, further efforts are required to enhance the assessment process. For instance, it is important to establish a clear goal and scope for HTA; adapt standardized methodologies for evaluating the performance of systematic reviews or meta-analyses; and provide comprehensive descriptions of the safety, clinical effectiveness, cost, and cost-effectiveness of the assessed technologies, thus improving the development of HTA in China.

**Supplementary Information:**

The online version contains supplementary material available at 10.1186/s41256-023-00339-6.

## Background

Health Technology Assessment (HTA) carefully assesses the costs and benefits of intervention to inform reimbursement and coverage decisions regarding how to allocate healthcare resources to different health technologies [1, 2]. HTA has played an increasingly important role on healthcare decision-making in China with the growth of HTA agencies and larger HTA research output since its introduction in the 1990s [3, 4]. However, the further development of HTA in China is confronted with potential challenges. Although it has been occasionally used by policy makers, it exists most often in the academic sector [5–9]. In 2016, Chen et al. conducted a study to understand the current development of HTA in China with the aim of identifying areas for improvement. This study identifies that overall HTA development in China is lower than that in developed countries [9].

From 2016 to 2021, HTA in China has gradually broadened from pure academic research to policy-or decision-oriented practices [6, 10, 11]. A milestone achievement for HTA in China, was the use of HTA evidence to update the National Reimbursement Drug List (NRDL) for the first time in 2017 [3, 12]. This was followed by the inclusion of anti-cancer drugs in the NRDL in 2018 and decision to routinize the use of HTA evidence in review processes in 2019 and 2020 by the National Healthcare Security Administration (NHSA) [11, 13]. In this period, however, some problems have become evident. For example, there is a lack of policy mechanisms for using HTA to support decision-making, insufficient HTA technical staff and expertise, and challenges regarding integrating multi-dimensional value assessment into evidence-informed deliberative processes [3]. In addition, researchers, and policy-maker’s understanding of HTA in China has gradually deepened, and the expectations for HTA development have increased.

Our primary aim is to determine what is the current development level of HTA in China, focusing on the NRDL process. Furthermore, how do the current progress and challenges compare to before 2017? To have a better understanding of the development level and potential challenges of HTA in China, we conducted this study to elicit diverse stakeholder views in China and compared it with the survey we conducted in 2016, thus informing policies and decisions to promote further development of HTA in China.

## Methods

### Study design and sample

This was a cross-sectional study in which an anonymous web-based survey was conducted using *Sojump* software from June to November, 2016, and February to April, 2021. The survey targeted policy makers, researchers and representatives of the pharmaceutical and medical device industry involved in HTA. Similar to the prior conducted 2016 survey, convenience sampling and snowball sampling were used to identify potential respondents. The process began by involving known HTA experts, who were then asked to recommend other stakeholders who met the above study criteria for participation. In addition, we searched the attendance lists of major HTA conferences in China and Chinese journals in the HTA field between 2013 and 2020.

### Survey development

In the distributed questionnaire, we incorporated the ‘Mapping of HTA Instrument’, Which has been employed to gauge respondents' perspectives on HTA development across seven middle-income countries (Argentina, Brazil, India, Indonesia, Malaysia, Mexico, and Russia) and three high-income countries (Australia, Canada, and the United Kingdom) [14]. The Mapping method adopted in this study was the same as that used in 2016, which is based on multiple HTA guidelines and standards that have been adopted broadly as well as discussions among many international HTA experts [9, 14]. The survey instrument was distinguished by two key elements: (i) the institutionalization of HTA and (ii) HTA process itself. Overall, the instrument comprises eight domains: (1) institutionalization of HTA; (2) identification; (3) priority setting; (4) assessment; (5) appraisal; (6) reporting; (7) dissemination of findings and conclusions; and (8) implementation in policy and practice.

For detailed information on the eight domains, their indicators, and the scoring methods of this instrument, please refer to Additional file 1: Figure S1. Respondents provided answers in either a dichotomous (YES/NO) or Likert scale format (0–3) to represent their views on each domain of HTA development. The maximum score for the eight domains is 146.

The questionnaire comprises two parts: the Mapping of HTA and demographic information. The demographic information section included items regarding the participant’s gender, age, region, years of work experience, educational background, highest degree obtained, and profession.

### Data collection

To reach a diverse range of participants, the survey link was shared with the target respondents through various channels, including email invitations, social media platforms such as WeChat (one of the most widely used Chinese social media platforms), and the professional network of the National Health Commission Key Laboratory of Health Technology Assessment [15]. Additionally, we distributed the link to stakeholders who were knowledgeable about HTA in China.

Before the main survey, cognitive pre-tests were conducted to ensure the face validity of the survey instrument and comprehensibility of the survey method. We conducted iterative offline pre-tests (n = 7) and after making the necessary revisions, we conducted additional online pre-tests (n = 29). During the pre-tests, the respondents were provided with test questions and encouraged to share feedback verbally.

Throughout the data collection process, the participants were explicitly informed about the voluntary nature of their involvement in the survey. They were given the freedom to decide whether to participate, and informed consent was obtained from all participants before completing the questionnaire. This ensured that their rights and privacy were respected throughout the research process. To ensure data quality, we cross-verified the dates provided by the participants with the actual survey dates to avoid inconsistencies or errors in the data.

### Data analysis

To mitigate the impact of confounding factors that could lead to outcome bias in the nonrandom assignment and enable a potential unbiased comparison, the Propensity Score Matching (PSM) methodology was typically applied [16, 17]. This method summarizes all relevant baseline characteristics into a single composite score, which can be used to determine whether there is sufficient overlap in characteristics between groups [18].

To minimize discrepancies and compare the mapping outcomes between the 2016 and 2021 groups, a 1:1 PSM was employed with a caliper size of 0.05 [16]. The matching algorithm included the following variables: years of work experience, profession, and educational background, which exhibited significant differences between the 2016 and 2021 groups in univariate analysis. Consequently, 183 cases from 2016 and 2021 were generated and used for further analyses. Descriptive analyses were conducted for all study variables.

Continuous variables were presented as means with standard deviations and were compared using Student *t*-test. Categorical variables were reported as frequencies and proportions and were compared using the chi-square test. Statistical significance was set at a two-sided *P*-value < 0.05. Statistical analyses were performed using R software (version 4.1.1) and Stata Statistics (version 14.2).

## Results

### Baseline characteristics

In total, 212 and 255 complete responses were obtained in 2016 and 2021, respectively. Detailed characteristics of the respondents in both groups are presented in Table 1. Prior to PSM, most respondents in 2016 were researchers (72.2%), resided in Eastern China (78.3%), and had educational backgrounds in medicine (51.9%). The most common highest degree obtained was a bachelor’s degree (47.2%). The average age of the respondents was 38.9 years (± 8.7 years), and the average years of work experience was 10.61 years (± 6.5 years).

**Table 1:** Characteristics of survey respondents before PSM

Variables	2016 group (n = 212)	2021 group (n = 255)
Gender		
Male	101 (47.60)	121 (47.50)
Female	111 (52.40)	134 (52.50)
Age (mean ± SD)	38.85 ± 8.71	37.76 ± 9.14
Region		
East China	166 (78.30)	220 (86.30)
Middle China	23 (10.80)	10 (3.90)
West China	16 (7.50)	19 (7.50)
Northeast China	4 (1.90)	4 (1.60)
Others^a^	3 (1.40)	2 (0.80)
Years of work experience (mean ± SD)	10.61 ± 6.50	7.92 ± 7.16
Educational background		
Medicine	110 (51.90)	147 (57.60)
Management	73 (34.40)	18 (7.10)
Economics	18 (8.50)	66 (25.90)
Others^b^	11 (5.20)	24 (9.40)
Highest degree obtained		
Doctoral	11 (5.20)	3 (1.20)
Master	39 (18.40)	23 (9.00)
Bachelor	100 (47.20)	123 (48.20)
Below bachelor	62 (29.20)	106 (41.60)
Profession		
Policy maker	33 (15.60)	52 (20.40)
Researcher	153 (72.20)	135 (52.90)
Industry-provider	26 (12.30)	68 (26.70)

^a^Others: including oversea regions

^b^Others: including low, education, or other major

Among the 255 respondents in the 2021 group, 52.9% were researchers. The majority of participants had an educational background in medicine (57.6%), and the most common highest degree obtained was a bachelor's degree (48.2%). Most respondents resided in Eastern China (86.3%). The average age of the respondents was 37.8 years (± 9.1 years), and the average years of work experience was 7.9 years (± 7.2 years). There were no significant differences regarding gender, age, or region between the two groups (*P* > 0.05).

The PSM process yielded a matched sample of 183 respondents in both the 2016 and 2021 groups, using a 1:1 nearest neighbor matching algorithm. Following PSM adjustment, no significant differences were observed between the two matched groups regarding the propensity score (generated by the PSM method, as shown in Additional file 2: Figure S2). This indicates that the baseline characteristics were effectively balanced between the two groups.

### Total score in each domain before PSM

As indicated in Table 2, the overall score for HTA development in China was higher in 2021 than in 2016 prior to applying PSM. Furthermore, the mean scores across various domains, including institutionalization, priority setting, assessment, appraisal, reporting, dissemination of findings and conclusions, and implementation in policy and practice., suggest notable progress in HTA development over time. However, the identification domain identified in 2021 had a relatively lower overall mean score than that in 2016.

**Table 2:** Score regarding the presence of the domains in the Mapping of HTA instrument in China before PSM

Domains	Max score	China (2016) n = 212		China (2021) n = 255	
		Score^**†**^	Normalized score (%)	Score^**†**^	Normalized score (%)
I. Institutionalization	28	16.84	60.14	18.63	66.54
II. Identification	19	3.47	18.26	3.31	17.42
III. Priority setting	18	10.23	56.83	11.05	61.39
IV. Assessment	39	31.22	80.05	32.09	82.28
V. Appraisal	9	4.57	50.78	4.76	52.89
VI. Reporting	11	6.52	59.27	7.94	72.18
VII. Dissemination of findings and conclusions	12	6.76	56.33	6.87	57.25
VIII. Implementation in policy and practice	10	4.35	43.50	4.73	47.30
Total	146	83.96	57.51	89.38	61.22

^†^Score = ∑(Each respondent’s total score of certain domain)/Number of respondents

### The detailed score in each domain after PSM

Within the “Institionization” domain, most respondents (63.4% in 2016 and 71.0% in 2021) indicated that China lacked an HTA agency that met the specified survey criteria. These criteria encompass aspects such as reporting to a Minister of Health/human resources or other relevant authorities, generating and/or endorsing HTA reports, and informing decisions regarding the introduction, reimbursement, and disinvestment of health technologies. Additionally, the respondents assessed the presence of essential elements for establishing a formal HTA program in China (see Table 3).

**Table 3:** Mean score regarding the main indicators in the Mapping of HTA Instrument and single-factor analysis results after PSM

Item	Mean score (SD)		Statistics^†^
	in 2016 (n = 183)	in 2021 (n = 183)	
I. Institutionalization (n = 116, in 2016; n = 130, in 2021)			
1. Interest in HTA expressed by government/policy makers which can be retrieved in official documents	1.78 (0.80)	2.22 (0.77)	− 4.455**
2. Commitment toward HTA from government/policy makers and it is expressed in official documents	1.38 (0.95)	1.63 (0.90)	− 2.134*
3. Public money (funding) is allocated to HTA as expressed in official documents	1.26 (0.92)	1.22 (0.94)	0.365
4. Willingness to commit public money (funding) to HTA as expressed in official documents	1.13 (0.98)	1.17 (0.93)	− 0.330
5. Support for HTA from several stakeholders as expressed in publicly available documents	1.90 (0.87)	2.27 (0.61)	− 3.856**
6. Organizational structure and institutional set-up in place	1.26 (0.90)	1.50 (1.04)	− 1.952
7. International network strategy available	1.76 (0.87)	1.85 (0.60)	− 0.988
8. Availability of human resource development	1.81 (0.82)	2.17 (0.88)	− 3.289**
II. Identification			
1. Monitoring system(s) to identify technologies in need of assessment in place	1.98 (1.02)	1.51 (1.10)	4.237**
2. Other activities involving identification are performed	1.99 (0.89)	1.98 (0.76)	0.127
III. Priority setting			
1. Explicit and transparent criteria and procedures	1.99 (0.91)	1.92 (0.68)	0.915
2. Process reflects the goals of the program	2.11 (0.86)	2.01 (0.76)	1.222
3. Stakeholder involvement is included	2.18 (0.95)	2.21 (0.77)	− 0.363
4. Information on priorities is set	2.08 (0.94)	1.89 (0.82)	2.144*
5. System(s) in place to review the international evidence base to set			
priorities	1.96 (0.77)	1.55 (0.66)	5.484**
6. Processes and outcomes of priority setting are evaluated	2.05 (0.85)	2.10 (0.65)	− 0.622
IV. Assessment			
1. Do the goal and scope of HTAs have a clear description of the following?			
(a) Healthcare problem(s)	1.80 (0.70)	1.55 (0.72)	3.472*
(b) Patient population	1.93 (0.72)	1.58 (0.75)	4.632**
(c) Practitioners or users	2.05 (0.76)	1.89 (0.70)	2.074*
(d) Healthcare setting(s)	1.92 (0.72)	1.88 (0.61)	0.630
2. Do HTAs include alternative technologies?			
(a) Description and technical characteristics of health technology under study, its alternatives, and current use	2.05 (0.53)	1.83 (0.45)	4.469**
3. Do HTAs assess the following?			
(a) Safety and clinical effectiveness	1.48 (0.58)	1.27 (0.48)	3.725^**^
(b) Cost and cost-effectiveness	1.72 (0.72)	1.49 (0.56)	3.391*
(c) Ethical analysis	2.02 (0.91)	2.12 (0.80)	− 1.102
(d) Organizational analysis	2.03 (0.92)	2.23 (0.72)	− 2.408*
(e) Social-cultural aspects	2.09 (1.03)	2.03 (1.01)	0.514
(f) Legal aspects	1.98 (0.86)	2.01 (0.63)	− 0.415
4. Do HTAs incorporate standardized methods?			
(a) Collection of new primary data	1.92 (0.73)	1.84 (0.94)	0.937
(b) Performance of systematic review or meta-analysis	1.84 (0.74)	1.56 (0.55)	4.017^**^
(c) Literature searches using key HTA databases	1.88 (0.72)	1.72 (0.55)	2.454*
(d) Classify and critically appraise the quality of the available studies	1.98 (0.75)	1.90 (0.51)	1.303
5. Do HTAs address generalizability and transferability?			
(a) Addressing generalizability and transferability	2.3 (0.56)	2.23 (0.66)	0.944
V. Appraisal			
1. Explicit, transparent, and replicable process	1.98 (0.86)	2.24 (0.63)	− 3.279*
2. Specification of stakeholder involvement	2.13 (0.90)	2.21 (0.73)	− 1.024
3. Mechanism(s) for appeal	2.06 (1.11)	1.90 (1.06)	1.397
VI. Reporting			
1.reporting			
(a) Use of guideline	2.28 (0.65)	2.34 (0.72)	− 0.912
2. Number of reports			
(a) Over the last year^‡^	2.93 (1.01)	2.70 (0.71)	2.564*
(b) Reports related to NRDL over the last year^‡^	3.09 (1.35)	3.09 (0.89)	0.000
VII. Dissemination of findings and conclusions			
1. Timeliness			
(a) HTA report is disseminated to decision makers before decision making	2.38 (0.82)	2.15 (0.79)	2.726*
2. Dissemination strategy			
(a) Content, target audience, and method of communication	2.08 (0.83)	2.28 (0.72)	− 2.493*
(b) Differentiate strategies for different subjects	2.08 (0.83)	2.34 (0.63)	− 3.325*
(c) Involvement of advisory groups	1.93 (0.77)	2.10 (0.68)	− 2.308*
VIII. Implementation in policy and practice			
1. Informing policy and practice			
(a) Existence of an administrative framework or link to regulatory process	2.42 (0.70)	2.37 (0.63)	0.628
(b) Availability of one or more implementation plans	2.42 (0.61)	2.30 (0.76)	1.746
2. Measuring HTA impact			
(a) System(s) in place to monitor and evaluate the impact of HTA	2.38 (0.92)	2.33 (0.97)	0.442

* < 0.05, ** < 0.001

^**†**^Statistics of Student t-test

^‡^The two indicators are different with other indicators in the scores (0–3), which range from 0 to 4

The mean score for ‘interest in HTA expressed by government/policy makers which can be retrieved in official documents’ (item I.1, *t* = −4.455,* P* < 0.05), ‘commitment toward HTA from government/policy makers and it is expressed in official documents’ (item I.2, *t* = −2.134,* P* < 0.05), ‘support for HTA from various stakeholders’ (item I.5, *t* = −3.856, *P* < 0.05), and ‘the availability of human resource development’ (item I.8, *t* = −3.289, *P* < 0.05) were significantly higher in 2021 than in 2016.

The ‘Identification’ domain focuses on the implementation of emerging technologies in need of assessment or those identified in the early monitoring system(s). The mean score of the indicator related to the existence of monitoring system(s) for emerging technologies (item II.1) is significantly higher in 2016 (1.98 out of 3) than in 2021(1.51 out of 3), (*t* = 4.237, *P* < 0.05). Regarding the performance of other activities involving identification (item II.2), the mean scores is 1.99 out of 3 in 2016 and 1.98 out of 3 in 2021.

Regarding the characteristics of China’s priority setting process, most indicators had a mean score of nearly 2 out of 3, demonstrating that these indicators were largely present. The indicator related to stakeholder involvement (item III.3) consistently received the highest score in both 2016 and 2021. However, respondents noted that the clarity of information on priorities (item III.4, *t* = 2.144, *P* < 0.05) and the extent of available literature (item III.5, *t* = 5.484, *P* < 0.05) were more explicit in 2016 than in 2021.

The ‘Assessment’ domain comprises 16 indicators categorized into five dimensions, inclusion of goal and scope, description of alternative technologies, aspects of assessment contents, standardized methods incorporation, and generalizability of the HTA scheme. The mean score of indicators concerning healthcare problems, patient population, and practitioners or users (item IV.1) was higher in 2016 than in 2021 (*P* < 0.05).

Regarding describing the technical characteristics of health technologies under study and their alternatives (item IV.2), the mean score in 2021 was lower than that in 2016 (*t* = 4.469, *P* < 0.05). Respondents reported that HTA activities in 2016 focused more on safety, clinical effectiveness, cost, and cost-effectiveness (item IV.3) than those in 2021 (*P* < 0.05). However, the organizational analysis showed the opposite trend (*t* = −2.408, *P* < 0.05).

Across all three indicators in the fourth dimension (Do HTAs incorporate standardized methods, item IV.4), the scores were below 2, indicating that this aspect of the HTA process was considered less developed by respondents. The indicator assessing whether HTA plans in China addressed generalizability and transferability (item IV.5) received the highest mean score among all 16 indicators in both 2016 (2.30 out of 3) and 2021 (2.23 out of 3).

The ‘Appraisal’ domain investigated whether a transparent and deliberative appraisal system, according to the participants, was in place. The respondents in 2021 believed that the appraisal process was more explicit, transparent, and replicable than those in 2016 (item V.1, *t* = −3.279, *P* < 0.05).

The ‘Reporting’ domain related to the utilization of the best practice guidelines in conducting and reporting HTA (item VI.1) received a score of 2.28 out of 3 in 2016 and 2.34 out of 3 in 2021, indicating that this aspect of HTA reporting was considered well developed in China over the five-year period.

Respondents reported a mean score of 2.93 out of 4 in 2016 and 2.70 out of 4 in 2021 for the number of HTA reports produced per year (item VI.2a, *t* = 2.564, *P* < 0.05), indicating a declining trend from 2016 to 2021. In contrast, the mean score for the number of HTA reports related to the NRDL per year (item VI.2b) was 3.09 out of 4 in both 2016 and 2021.

The ‘Dissemination of findings and conclusions’ domain related to the timeless of HTA report dissemination to decision makers and some dissemination strategies. It is worth noting that all indicators related to dissemination strategies (item VII.2) in 2021 showed significant improvement compared to those for 2016 (*P* < 0.05).

The ‘Implementation in policy and practice’ domain is relevant to the policy and practice information provision and the HTA impact measurement. The mean scores for each indicator were above 2 out of 3, showing that these indicators were largely present. These findings suggest that HTA implementation in policy and practice is moderately well developed in China.

Overall, the level of HTA development in China was higher than that in middle-income countries (Argentina, Brazil, India, Indonesia, Malaysia, Mexico, and Russia) and lower than that in high-income countries (Australia, Canada, and the United Kingdom) in 2016 and 2021 (see Additional file 3: Table S1). In our previous research conducted in 2016, we found that China scored lower than all ten countries regarding institutionalization level based on the views of survey respondents. However, the current results indicate that China has made significant advancements in institutionalization, although in 2021 the score for China is still lower than that for middle-income countries.

## Discussion

In this study, we employed HTA mapping instruments to gather diverse stakeholder perspectives in China during in 2016 and 2021. By comparing the mapping outcomes between these two groups using PSM, we gained valuable insights into the current state of HTA and the progress made in HTA development in China from 2016 to 2021. In addition, this study can also provide practical insights for the ‘selected countries’ [14], identifying the benefits of evaluating their HTA development after three to five years, especially in those countries that have announced healthcare reforms and changes to their HTA processes [19–21]. This evaluation can assist various stakeholders, including governments, HTA organizations, industry players, and other relevant actors in assessing HTA development at the country level. Furthermore, the study results provide valuable information to inform HTA strategies and justify investments in HTA.

Overall, the mean scores for most domains in 2021 were higher than those in 2016, indicating an improvement in HTA development. However, it is worth noting that the assessment and implementation domain have lower scores in 2021 than in 2016. Although China scored lower than the three developed countries, its overall HTA development score was comparable to those of the ten countries studied. This recognition signifies the significant achievements that China has made in HTA development since 2016.

Although there has been a significant improvement in China’s level of institutionalization in 2021 compared to 2016, our findings indicate that it still lags behind other countries. While HTA in China has recently expanded from pure academic research to include policy or decision-oriented practices, there is remains a lack of well-established systems for integrating HTA evidence into advisory, pricing, and reimbursement decision-making processes within health administration and payer organizations in China [11, 13, 22, 23]. In addition to China, the process of HTA institutionalization in other low- and middle-income countries remains immature [24, 25]. The insufficient institutionalization of HTA hinders its further development and utilization. It is crucial to prioritize national-level HTA institutionalization to promote the continued advancement. The key aspects of this institutionalization process include the identification and prioritization of HTA issues, adequate funding for HTA activities and human resources, establishment of implementation standards for various health technologies, development of HTA appraisal guidelines, and implementation of robust quality control mechanisms for HTA [6, 11, 19].

The scores for most indicators in the assessment domain were lower in 2021 than in 2016, potentially indicating the declining quality and validity of HTA evidence. Although HTA development in China has made significant strides in utilizing HTA evidence to inform policy decisions and successfully update the NRDL, it is important to acknowledge that most HTA studies in China are sponsored by manufacturers [11, 26]. This raises concerns about potential conflicts of interest and uncertainties regarding the quality of the submitted cost and cost-effectiveness analysis (CEA) evidence. For example, Xie et al. have discovered that industry-sponsored CEAs are significantly more likely to report incremental cost-effectiveness ratios below commonly used thresholds than CEAs lacking such sponsorship [27]. To mitigate the potential influence of the utilization of pharmacoeconomic evidence provided by the industry, it is imperative for concerned institutions to establish and endorse standardized procedures for the disclosure of Financial Conflicts of Interest (FCoI) in pharmacoeconomic evaluations. This should encompass the comprehensive FCoI status disclosure of the input parameters’ sources. When submitting relevant studies, the mandatory inclusion of existing disclosure forms, such as the International Committee of Medical Journal Editors (ICMJE) disclosure form, should be enforced to enhance transparency.

Furthermore, even when relevant evidence is available, the adoption of appropriate methodologies is crucial for generating meaningful results that support decision making. Although global resources and best practices can be leveraged and used, China still faces challenges in adapting methodological approaches to meet national or local needs [11]. This is particularly true when applying standard HTA methodologies to assess the efficacy and cost-effectiveness of traditional Chinese medicine, which may require special considerations [11, 26]. According to the top 10 challenges identified by The International Network of Agencies for Health Technology Assessment (INAHTA), there is a perception from INAHTA member agencies that the quality of evidence is declining, with fewer randomized trials being conducted and more observational and real-world data being used [24]*.*

The development score for an explicit, transparent, and replicable appraisal process improved in 2021 compared to that in 2016. Notably, in 2017, a significant change was implemented in the criteria for determining the inclusion of drugs in the NRDL, whereby both clinical and economic evaluations were stipulated as requirements for inclusion [3, 12]. This shift from qualitative expert consensus to quantitative evidence has led to substantial progress in value-based price negotiations for price and reimbursement decisions [3]. Furthermore, the NHSA has provided guidelines and material requirements to pharmaceutical companies to ensure the provision of comprehensive dossiers supporting the inclusion of their products in the NRDL [28–30]. This measure enhances the explicit, transparent, and replicable appraisal processes.

This study had some limitations. First, the generalizability of the findings may be limited due to the convenience sampling approach. It was not possible to identify the statistics of the target population of HTA stakeholders in China. Therefore, the representativeness of our sample could not be fully assessed. Second, the questionnaire was adapted from an international study on the mapping levels of HTA development. There may have been imprecision and misinterpretation when translating the mapping instrument from English to Chinese. Third, within the policy-maker and researcher group, owing to their different levels of education, understanding, and application of HTA, the attached meaning to responses may vary across respondents.

## Conclusions

From the perspective of multiple stakeholders, HTA development in China has made significant progress from 2016 to 2021, however, more efforts should be given to the assessment process ensuring a higher quality of HTA evidence before conducting HTA activity. Further qualitative research such as in-depth interviews and focus group discussions with multiple stakeholders, including HTA researchers and policy makers, is required to determine the specific reasons that influence the development level of HTA in China.

### Supplementary Information

**Additional file 1: Figure S1.** The Mapping of HTA instrument. *Note* This instrument was developed by Wija Oortwijn et al. This figure contains the detailed eight domains and their indicators as well as the scores (max. total score 146).

**Additional file 2: Figure S2.** Common support test. *Note* After PSM adjustment, there were no significant differences between the two matched groups with regard to the propensity score, that is, baseline characteristics were balanced.

**Additional file 3. Table S1.** Level of HTA development per domain in the selected middle-and high-income countries. *Note* Data of the seven middle-income and three high-income countries are from the research of Oortwijn et al. [14]. Middle-income countries include Argentina, Brazil, India, Indonesia, Malaysia, Mexico, and Russia, high-income countries include Australia, Canada, and the United Kingdom.

## Data Availability

The data will be shared on reasonable request to the corresponding author.

## Abbreviations

HTA: Health Technology Assessment
NRDL: National Reimbursement Drug List
NHSA: National Healthcare Security Administration
PSM: Propensity Score Matching
CEA: Cost-Effectiveness Analysis
FCoI: Financial Conflicts of Interest
ICMJE: International Committee of Medical Journal Editors
INAHTA: The International Network of Agencies for Health Technology Assessment
