# Supplementary figures and images for: Mapping of health technology assessment in China: a comparative study between 2016 and 2021
Source: Glob Health Res Policy. 2024 Jan 16;9:4. doi: 10.1186/s41256-023-00339-6 (PMC10790493; doi:10.1186/s41256-023-00339-6)

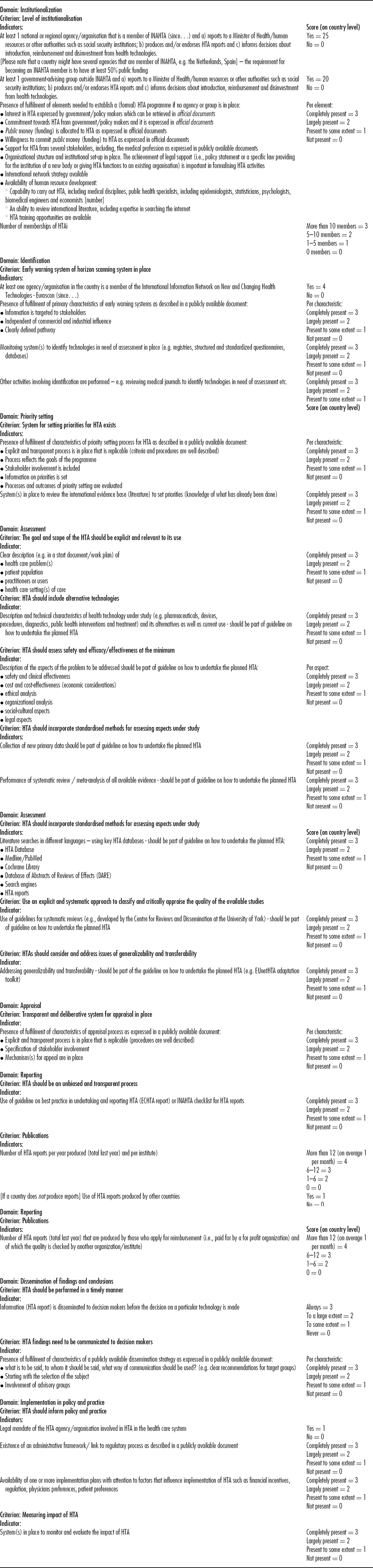

Supplement: Supplementary file 1 — Additional file 1: Figure S1. The Mapping of HTA instrument. Note This instrument was developed by Wija Oortwijn et al. This figure contains the detailed eight domains and their indicators as well as the scores (max. total score 146). [file 41256_2023_339_MOESM1_ESM.gif]

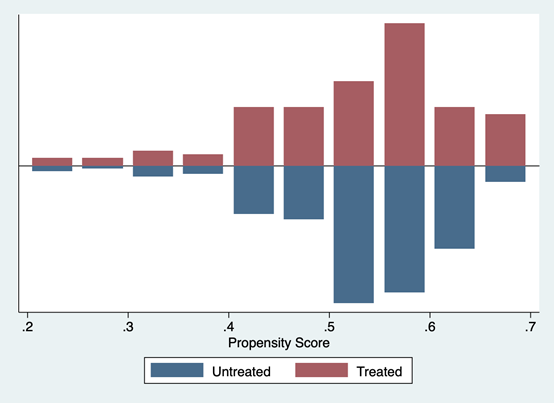

Supplement: Supplementary file 2 — Additional file 2: Figure S2. Common support test. Note After PSM adjustment, there were no significant differences between the two matched groups with regard to the propensity score, that is, baseline characteristics were balanced. [file 41256_2023_339_MOESM2_ESM.jpg]
