# Supplementary material for: Mapping of health technology assessment in China: a comparative study between 2016 and 2021
Source: Glob Health Res Policy. 2024 Jan 16;9:4. doi: 10.1186/s41256-023-00339-6 (PMC10790493; doi:10.1186/s41256-023-00339-6)
Supplement: Supplementary file 3 — Additional file 3. Table S1. Level of HTA development per domain in the selected middle-and high-income countries. Note Data of the seven middle-income and three high-income countries are from the research of Oortwijn et al. [14]. Middle-income countries include Argentina, Brazil, India, Indonesia, Malaysia, Mexico, and Russia, high-income countries include Australia, Canada, and the United Kingdom. [file 41256_2023_339_MOESM3_ESM.docx]

**Supplementary Table 1 Score regarding the presence of the domains in the Mapping of HTA instrument in some middle- and high-income countries**

| **Domains** | **Max Score** | **Middle-income countries** | | **High-income**  **countries** | |
| --- | --- | --- | --- | --- | --- |
|  |  | Score | Normalized Score (%) | Score | Normalized Score (%) |
| I. Institutionalization | 28 | 18.9 | 67.3 | 28.0 | 100.0 |
| II. Identification | 19 | 1.9 | 9.8 | 12.3 | 64.9 |
| III. Priority setting | 18 | 7.1 | 39.7 | 13.7 | 75.9 |
| IV. Assessment | 39 | 15.6 | 39.9 | 28.3 | 72.6 |
| V. Appraisal | 9 | 1.9 | 20.6 | 7.3 | 81.5 |
| VI. Reporting | 11 | 5.7 | 51.9 | 11.0 | 100.0 |
| VII. Dissemination of findings and conclusions | 12 | 3.7 | 31.0 | 9.0 | 75.0 |
| VIII. Implementation in policy and practice | 10 | 3.1 | 31.4 | 7.3 | 75.3 |
| Total | 146 | 57.9 | 39.6 | 116.9 | 80.1 |

Note. Data of the seven middle-income and three high-income countries are from the research of Oortwijn et al (Reference 14). Middle-income countries include Argentina, Brazil, India, Indonesia, Malaysia, Mexico, and Russia, high-income countries include Australia, Canada, and the United Kingdom.
